# Isolate-Based Surveillance of *Bordetella pertussis*, Austria, 2018–2020

**DOI:** 10.3201/eid2703.202314

**Published:** 2021-03

**Authors:** Adriana Cabal, Daniela Schmid, Markus Hell, Ali Chakeri, Elisabeth Mustafa-Korninger, Alexandra Wojna, Anna Stöger, Johannes Möst, Eva Leitner, Patrick Hyden, Thomas Rattei, Adele Habington, Ursula Wiedermann, Franz Allerberger, Werner Ruppitsch

**Affiliations:** Institute for Medical Microbiology and Hygiene, Austrian Agency for Health and Food Safety, Vienna, Austria (A. Cabal, D. Schmid, A. Chakeri, A. Stöger, F. Allerberger, W. Ruppitsch);; MEDILAB, Teaching Laboratory of the Paracelsus Medical University, Salzburg, Austria (M. Hell, E. Mustafa-Korninger, A. Wojna);; Centre for Public Health, Medical University Vienna, Vienna (A. Chakeri);; MB-LAB Clinical Microbiology Laboratory, Innsbruck, Austria (J. Möst);; Consultant Laboratory for Bordetella of the Robert Koch Institute, Medical University of Graz, Graz, Austria (E. Leitner);; Centre for Microbiology and Environmental Systems Science, University of Vienna, Vienna (P. Hyden, T. Rattei);; Children’s Health Ireland at Crumlin, Dublin, Ireland (A. Habington);; Institute of Specific Prophylaxis and Tropical Medicine, Medical University of Vienna, Vienna (U. Wiedermann)

**Keywords:** Bordetella pertussis, vaccine-preventable diseases, acellular vaccines, core-genome multilocus sequence typing, cgMLST, clusters, Austria, surveillance, vaccines, bacteria, respiratory infections

## Abstract

Pertussis is a vaccine-preventable disease, and its recent resurgence might be attributable to the emergence of strains that differ genetically from the vaccine strain. We describe a novel pertussis isolate-based surveillance system and a core genome multilocus sequence typing scheme to assess *Bordetella pertussis* genetic variability and investigate the increased incidence of pertussis in Austria. During 2018–2020, we obtained 123 *B. pertussis* isolates and typed them with the new scheme (2,983 targets and preliminary cluster threshold of <6 alleles). *B. pertussis* isolates in Austria differed genetically from the vaccine strain, both in their core genomes and in their vaccine antigen genes; 31.7% of the isolates were pertactin-deficient. We detected 8 clusters, 1 of them with pertactin-deficient isolates and possibly part of a local outbreak. National expansion of the isolate-based surveillance system is needed to implement pertussis-control strategies.

*Bordetella pertussis* is the main causative agent of the reemerging respiratory disease commonly known as whooping cough (*1*). *B. pertussis* infection usually affects infants, toddlers, and children of school age, although adolescents and adults also can get infected and have symptoms. In addition, because transmission of pertussis can go unnoticed, asymptomatic carriers are considered an important source of infection (*2*). Despite its low sensitivity, culturing pertussis from nasopharyngeal swabs remains the standard diagnostic technique, although today it is scarcely performed (*3*).

To some extent, pertussis can be prevented by vaccination with either cellular or acellular vaccines (*4*). In Austria, cellular pertussis vaccines were replaced in 1998 by acellular vaccines (ACVs), and ever since, either 2 (filamentous hemagglutinin [FHA] and pertussis toxin [PTX]) or 3 (FHA, PTX, and pertactin) component vaccines have been used for primary vaccination. Booster immunizations have been recommended since 2003 for children of school age and adolescents. These vaccines include either 3 (FHA, PTX, and pertactin) or 5 (FHA, PTX, pertactin, FIM2, and FIM3) components. 

Despite vaccinations, the incidence of pertussis has been increasing in the past few decades (*5*–*7*). In Austria, 579 pertussis cases were reported in 2015; the number number increased to 1,274 in 2016, 1,411 in 2017, 2,198 in 2018, and 2,246 in 2019. The increase in the incidence of pertussis worldwide can be explained partially by the loss of the protective effect after immunity wanes; this loss is strongly associated with ACV use (*8*). Another factor that contributes to the resurgence of pertussis is the emergence of vaccine-evasive *B. pertussis* strains that differ genetically from the vaccine strains (*9*,*10*).

A molecular study conducted on pertussis cases from 3 different cities in Austria assessed the genetic variability of *B. pertussis* nationwide (*5*). However, the study used respiratory samples to perform PCR, followed by Sanger sequencing; therefore, typing was not based on whole-genome sequencing (WGS) of *B. pertussis* isolates.

Because of the rise in the incidence of pertussis in Austria in recent years, we investigated pertussis cases from 3 states in Austria to assess the genetic variability of their *B. pertussis* isolates through WGS-based typing. The first objective was to set up a national isolate-based surveillance system, complementary to the case-based surveillance system in Austria, for collecting isolates from patients with suspected pertussis. Second, we aimed to characterize and to compare *B. pertussis* isolates with the vaccine strain Tohama I and other isolates from different geographic regions outside Austria.

## Methods

### Setup of the Surveillance System and Sequencing

**Figure 1:**
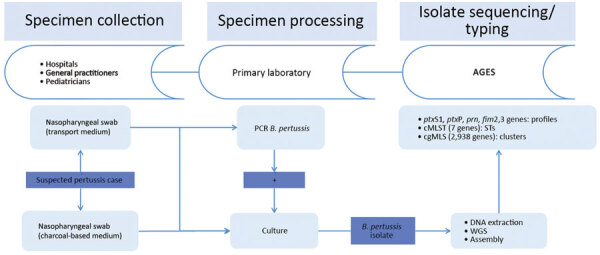
Flow chart of the *Bordetella pertussis* isolate–based surveillance system, Austria, May 2018–May 2020. AGES, Agentur für Gesundheit und Ernährungssicherheit (Austrian Agency for Health and Food Safety); cgMLST, core-genome multilocus sequence typing; ST, sequence type.

For 2 years (May 2018–May 2020), hospitals, general practitioners, and pediatricians using clinical laboratories located in 3 states in Austria (Salzburg, Tyrol, and Styria) were asked to collect >1 nasopharyngeal swab containing transport medium (ESwab; Copan, https://www.copangroup.com) from patients with suspected *B. pertussis* infection (Figure 1). When possible, a second nasopharyngeal swab containing charcoal-based medium (Transystem Amies medium with charcoal; Copan) was collected.The swabs were then sent to the clinical laboratory of each state participating in the study. For each suspected case, PCR was performed using the swab containing the transport medium with a commercial kit (BD MAX, Becton Dickinson, http://bd.com; or BORDETELLA R-gene, bioMérieux, https://www.biomerieux.com). When PCR results were positive, either the same swab used for PCR or, if available, the charcoal swab was stroked on Oxoid *Bordetella*–selective medium (Thermo Fisher Scientific, https://www.thermofisher.com) or Bordet Gengou agar with 15% sheep blood (Becton Dickinson), followed by cultivation at 37°C under aerobic and humid conditions for 48–120 hours. Colonies compatible with *B. pertussis* were tested by MALDI Biotyper software version 3.0 (Bruker, https://www.bruker.com) or VitekMS software version 3.2 (bioMérieux). Colonies indentified as *B. pertussis* were sent to the Austrian Agency for Health and Food Safety in Vienna for further DNA extraction and 300-bp paired-end WGS using an Illumina Miseq device (https://www.illumina.com), as described in Appendix 1. Additional information on the sequencing process, de novo assembly, and sequence quality checks also are found in Appendix 1. The Illumina reads of the 123 isolates in Austria have been deposited in the National Center for Biotechnology Information (NCBI) Sequence Read Archive repository under project number PRJNA642701.

### Generation of a *B. pertussis* cgMLST Scheme

A stable, ad hoc, core-genome multilocus sequence typing (cgMLST) scheme and accessory genome scheme were created by using Ridom SeqSphere + version 4.1.9 (Ridom, https://www.ridom.de; Appendix 1). In brief, 15 genomes (Appendix 2 Table 1) were used as query genomes and the Tohama I vaccine strain genome (GenBank accession no. NC_002929.2) as a seed genome. Afterwards, 263 taxonomic and quality outliers were discarded, leaving a total of 2,983 core genome targets (Appendix 2 Table 2) and 179 accessory genome targets (Appendix 2 Table 3). We considered as core genes only those targets (i.e., genes) that were present in 100% of the genomes. Further validation of the scheme was based on a selection of *B. pertussis* genomes available in NCBI (n = 391), many of which were associated with outbreaks (Appendix 2 Table 4), and an old collection of clinical *Bordetella* sp*.* strains from Austria (Appendix 2 Table 5).

### Typing of *B. pertussis* Isolates and Comparative Analysis

During the 2-year study period, all the clinical isolates collected within the isolate-based surveillance system were typed with our newly implemented cgMLST scheme. Allelic differences among the isolates from Austria and the vaccine strain Tohama I were visualized by generating minimum spanning trees with a preliminary cluster threshold established at <6 alleles (Appendix 1). We extracted the sequence types (STs) from the WGS data corresponding to the classical multilocus sequence typing (*11*), the variants and mutations present in each of the genes used as vaccine antigens (*ptx*S1, *ptx*P, *prn*, *fim*2, *fim*3), and their combination (genetic profiles).

To be certain that our scheme could be applied beyond our set of *B. pertussis* isolates from Austria, we used a selection (n = 106) of *B. pertussis* genomes, including outbreak strains used in the validation of the cgMLST scheme, to perform a genomic comparative analysis (Appendix 1; Appendix 2 Table 4). We compared the gene content obtained for our cgMLST scheme with the cgMLST scheme developed by the Pasteur Institute (Paris, France) (*12*). In addition, we compared the results obtained when applying our cgMLST with those derived from a single-nucleotide polymorphism (SNP)–based analysis on the 123 isolates from Austria (Appendix 1).

### Statistical Analysis

Personal information and vaccination status were obtained for each pertussis culture-positive case-patient from the national electronic reporting system. We calculated odds ratios with Stata software version 13 (StataCorp, https://www.stata.com) to measure for associations between pertactin deficiency and vaccination status. In the analysis, we included all case-patients who had received >1 dose of pertussis vaccine and those reported as unvaccinated. Case-patients with an unknown vaccination status (n = 31) were excluded from the analysis. Statistical significance was defined as p<0.05 by using the Pearson χ^2^ test or Fisher exact test.

## Results

### Culture-Positive Cases

At the Austrian Agency for Health and Food Safety, we received 123 *B. pertussis* isolates, collected from 123 pertussis case-patients (Table 1), through our newly implemented isolate-based pertussis surveillance system during May 2018–May 2020. Fewer than 20% of the total pertussis cases reported in Salzburg state (n = 310) in 2018 were estimated to be culture-positive, and no information on the proportion of cases with a positive pertussis culture was available for the other 8 states in Austria.

**Table 1:** Demographic characteristics of the 123 pertussis case-patients in the *Bordetella pertussis* isolate–based surveillance system, Austria, May 2018–May 2020

Characteristic	No.
Age group, y	
<1	8
1–4	15
5–9	31
10–14	31
15–19	7
20–29	3
30–39	7
40–49	11
50–59	3
>60	7
Sex	
F	69
M	54
State	
Salzburg	86
Tyrol	21
Styria	12
Upper Austria	4
Clinical symptoms	
Coughing fits	66
Cough >4 weeks	64
Medical whooping cough diagnosis	37
Missing data	7
Post-coughing vomiting	7
Inspiratory whooping	5
Asymptomatic	2
Vaccination status	
Vaccinated	53
1st booster	1
2nd booster	22
3rd booster	16
4th booster	7
Unknown doses	7
Not vaccinated	39
Unknown	31

**Figure 2:**
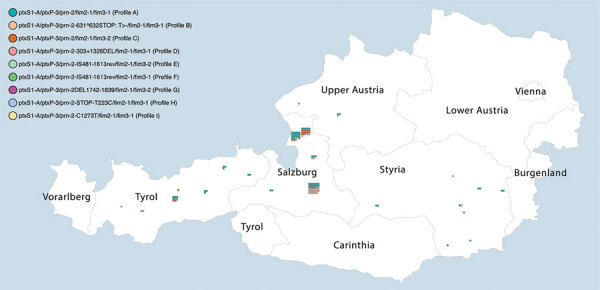
Pertussis cases by district of residence of case-patient and genetic profile of the corresponding *Bordetella pertussis* isolate identified in a *B. pertussis* isolate–based surveillance study, Austria, May 2018–May 2020. Each dot represents 1 case. Cases grouping next to each other belong to the same district. To protect patient confidentiality, only states and not districts are labeled.

A total of 119 *B. pertussis* isolates belonged to patients with PCR-positive confirmed pertussis from Salzburg, Tyrol, and Styria (Figure 2), and 4 isolates belonged to pertussis case-patients identified in the state of Upper Austria, provided by a clinical microbiology laboratory located in Salzburg (MB-LAB Clinical Microbiology Laboratory). Overall, 15 *B. pertussis* isolates belonged to culture-positive pertussis case-patients who lived in the same household with >1 other culture-positive case-patient. Additional metadata for the 123 pertussis cases are presented in Appendix 2 Table 6.

### Sequence Types and Typing of Vaccine Target Genes

**Figure 3:**
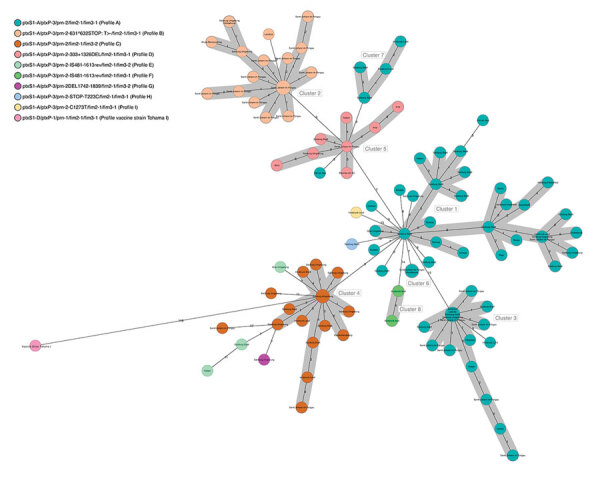
Minimum spanning tree of 123 *Bordetella pertussis* isolates and their clusters by genetic profile based on core-genome multilocus sequence typing in a *B. pertussis* isolate–based surveillance study, Austria, May 2018–May 2020. Numbers on connection lines represent the number of allelic differences among the isolates.

The *B. pertussis* isolates obtained from the 123 pertussis patients in Austria differed in sequence type and in the vaccine antigen genes from the vaccine strain Tohama I (Table 2). We detected ST2 for all but 1 isolate, which was of ST83. We found 9 different genetic profiles (A–I), 1 of which was new (profile G) (Table 2; Figure 3; Appendix 2 Table 7).

**Table 2:** Genetic profiles of the 123 *Bordetella pertussis* isolates obtained through the *B. pertussis* isolate–based surveillance system, Austria, May 2018–May 2020*

Profile	No. (%)	*B. pertussis* vaccine antigen genes					Reference
		*ptx*S1	*ptx*P	*prn*	*fim*2	*fim*3	
Profile vaccine strain Tohama I	0	*ptx*S1-D	*ptx*P-1	*prn*-1	*fim*2-1	*fim*3-1	NA
Profile A	64 (52.3)	*ptx*S1-A	*ptx*P-3	*prn*-2	*fim*2-1	*fim*3-1	(*13*,*14*)
Profile B	23 (18.7)	*ptx*S1-A	*ptx*P-3	*prn*-2–631^632STOP:T>-	*fim*2-1	*fim*3-1	(*15*)
Profile C	20 (16.2)	*ptx*S1-A	*ptx*P-3	*prn*-2	*fim*2-1	*fim*3-2	(*13*,*14*)
Profile D	8 (6.50)	*ptx*S1-A	*ptx*P-3	*prn*-2–303+1326DEL	*fim*2-1	*fim*3-1	(*16*)
Profile E	3† (2.44)	*ptx*S1-A	*ptx*P-3	*prn*-2-IS481–1613rev	*fim*2-1	*fim*3-2	(*13*,*14*)
Profile F	2 (1.62)	*ptx*S1-A	*ptx*P-3	*prn*-2-IS481–1613rev	*fim*2-1	*fim*3-1	(*14*)
Profile G	1 (0.8)	*ptx*S1-A	*ptx*P-3	*prn*-2- DEL1742–1839‡	*fim*2-1	*fim*3-2	This study
Profile H	1 (0.8)	*ptx*S1-A	*ptx*P-3	*prn*-2-STOP-T223C	*fim*2-1	*fim*3-1	(*14*)
Profile I	1 (0.8)	*ptx*S1-A	*ptx*P-3	*prn*-2-C1273T	*fim*2-1	*fim*3-1	(*13*,*14*)

*NA, not applicable. †One isolate was sequence type 83. ‡This isolate showed an insertion longer than 200 bp in the *prn* gene combined with a partial deletion at positions nt 1742–1839.

We found 7 pertactin-deficient profiles (B and D–I), representing 31.7% (n = 39) of the isolates, and 6 different known pertactin inactivation mechanisms (*13*–*16*; Table 2; Appendix 2 Table 7). Pertactin-deficient isolates were mostly of profile B (n = 23 [18.7%]). Twenty case-patients (51.3%) with pertactin-deficient isolates had been vaccinated, 11 (28.2%) case-patients were unvaccinated, and for 8 (20.5%) case-patients, vaccination status was unknown. Case-patients having received >1 dose of pertussis ACV were 1.5 times more likely to have a pertactin-deficient *B. pertussis* isolate (of any genetic profile) compared with unvaccinated case-patients, although this relationship was not statistically significant (unadjusted odds ratio 1.5, 95% CI 0.6–3.8). Persons living in the district of St. Johann in Pongau (Salzburg state) were 21.17 (95% CI 6.7–81.1) times more likely to have profile B, and this association was significant (p<0.001). Stratifying by vaccination status, vaccinated persons from St. Johann in Pongau were 13.3 (95% C: 2.9–99.1; p<0.001) times more likely to present profile B, whereas unvaccinated ones had 58.5 (95% CI 5.6–1876; p<0.001) times more chances to present profile B. No association was seen between the different age groups or having a pertactin-deficient profile and having profile B.

### cgMLST and Comparative Analysis

**Figure 4:**
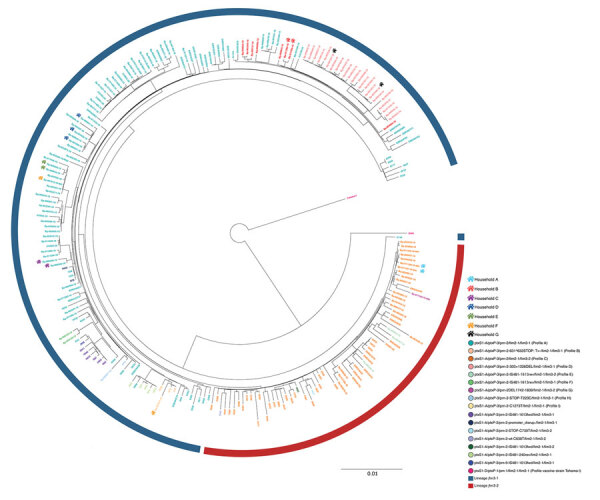
Maximum-likelihood phylogenetic tree generated using core-genome multilocus sequence typing data from 106 outbreak genome sequences from the United States and the United Kingdom and the 123 *Bordetella pertussis* isolates identified in an isolate-based surveillance study, Austria, May 2018–May 2020. Isolate identifiers are colored by genetic profile. These genetic profiles include profiles A–I, defined in this study, and other genetic profiles described outside of Austria. The circular blue line represents isolates of *fim*3-1 lineage; the circular red line represents *fim*3-1 isolates. A color-coded house-like symbol indicates isolates obtained from case-patients living in the same household. Scale bar indicates nucleotide substitutions per site.

The 123 *B. pertussis* isolates were closely related, differing by a maximum of 38 alleles (<44 alleles when including the accessory genome), and they seemed to cluster in groups (Figure 3). We observed that we could separate *fim*3-1 and *fim*3-1 isolates into 2 branches (Figure 4) and that pertactin-deficient isolates of genetic profile B grouped together. Isolates of profile B (n = 23) differed by a maximum of 9 alleles when including the only isolate from Tyrol of that profile, and by <6 alleles, excluding the isolate from Tyrol. All other isolates were from Salzburg (n = 20), Styria (n = 1), and Upper Austria (n = 1). Isolates from profile D also clustered together (<6 alleles), differing by >8 alleles with isolates of profiles A and B.

With the preliminary cluster threshold of <6 alleles, we distinguished 8 clusters (Figure 3; Appendix 1). Cluster 1 integrated only profile A isolates (n = 26) from all states. Isolates in cluster 2 (n = 22) were of profile B. Most of the isolates in this cluster (n = 20) originated from case-patients living in Salzburg state. Eighteen of them resided in the district of St. Johann in Pongau. Of the 2 case-patients who did not live in Salzburg state, 1 had a confirmed epidemiologic link with a pertussis-positive relative in Salzburg. A trend compatible with a local outbreak of genetic profile B *B. pertussis* was distinguished (Appendix 1 Figure 1) for this cluster when we compared the number of profile B cases with the total number of reported cases (culture positive and nonculture positive) in the same period for St. Johann in Pongau (n = 160). In addition, a peak of cases in November 2018 corresponded to a small peak of cases with genetic profile B isolates. Cluster 3 had 19 isolates of profile A that were obtained from case-patients living in Salzburg, Tyrol, and Styria. Cluster 4 had 18 isolates of profile C from Salzburg, Tyrol, and Styria. Cluster 5 consisted of 9 profile D isolates from Salzburg, Styria, Tyrol, and Upper Austria. Cluster 6 included 5 profile A isolates from Salzburg and Upper Austria, and cluster 7 combined 4 profile A isolates from Salzburg and Tyrol. Cluster 8 had only 2 isolates, both of profile F, which originated from case-patients from Tyrol. Isolates from profiles E, G, H, and I did not cluster with any other isolate. The ST83 isolate did not cluster with any other isolate and differed from isolates in cluster 4 by >9 alleles.

In 6 households (A–E and G) (Appendix 2 Table 8), we confirmed the transmission of the same *B. pertussis* strain between 2 or 3 household members with a maximum of 4 allelic differences and an identical genetic profile. In 1 household (F), transmission of *B. pertussis* was ruled out when cgMLST revealed 18 allelic differences between 2 isolates obtained from 2 case-patients living together, each of them with a *B. pertussis* strain of a different genetic profile (A and I).

We developed a comparative analysis between our 123 *B. pertussis* isolates and 106 *B. pertussis* genomes from NCBI, including mostly isolates from the United States and United Kingdom (Appendix 2 Table 4) from the 2010 and 2012 epidemics (*13*,*14*,*16*) (Figure 4). All *fim*3-1 isolates were clearly separated from *fim*3-2 isolates, but we could also differentiate isolates from the same country in different branches. On the basis of the *prn* type, which comprised the *prn* wild type alleles or mutations in this gene (i.e., insertions, deletions, and truncations), we distinguished isolates grouping closely to each other, sometimes originating from different countries. Genetic profiles A, C, H, and I were also represented in strains from outside Austria.

We compared target content between the Pasteur Institute’s cgMLST scheme and ours (Appendix 2 Table 9). A total of 1,749 genes were common to both schemes; 1,239 genes were only present in our scheme, and 294 genes were only present in Pasteur Institute’s scheme. The SNP analysis revealed isolates grouping in clusters in a similar way to that resulting from the cgMLST analysis (Appendix 1 Figure 2).

## Discussion

Our newly implemented *B. pertussis* isolate–based surveillance system has contributed to a better understanding of the molecular epidemiology of pertussis in Austria. No relationship existed between the pertussis incidence in the 3 states in Austria and the number of isolates collected, which depended mainly on the expertise of the respective laboratories in obtaining *B. pertussis* cultures. The estimated proportion (≈20%) of culture-positive pertussis cases found in relation to the total number of pertussis cases reported in Salzburg state was consistent with previous reports (*17*), whereas other authors reported up to 30% (*18*).

Results retrieved from the typing of the 123 *B. pertussis* isolates revealed the presence of the *ptxP*-3 allele in all isolates tested, consistent with other studies (*19*–*21*), thereby clearly indicating a divergence from the vaccine strain in Austria and a substitution among currently circulating *B. pertussis* strains of the *pt*xP-1 allele by the *ptxP*-3 allele. In comparison, during 2002–2008, a previous study in Austria still detected the *ptxP*-1 allele in 7% of the samples (*5*). Previous data also showed most of *B. pertussis* isolates (≈80%) grouping in the *fim*3-1 clade, which is more ancestral than *fim*3-2 (*22*). Therefore, not surprisingly, the genetic profile A (*fim3*-1 clade) was one of the most frequently detected genetic profiles globally (*22**–**24*), consistent with the findings of our study.

As for the proportion of pertactin-deficient isolates detected (31.7%), this finding was similar to the frequency reported during 2012–2015 in Norway, where ACVs were also introduced in 1998 and booster doses recommended after 2001 (*15*). In contrast, up to 98% of pertactin-deficient isolates were reported outside the European Union (*25*). In general, the proportion of *B. pertussis* isolates with pertactin deficiency seemed to be vary among countries depending on the vaccination schedule and vaccine type used (*26*). Those countries still using cellular pertussis vaccines have never or rarely reported pertactin-deficient isolates (*27*,*28*), whereas countries using ACVs have seen a direct association between the year of introduction of ACVs in the country and the appearance of pertactin-deficient isolates (*15*). Moreover, ACV-vaccinated persons seem more susceptible to pertactin-deficient strains than to pertactin-producing strains, given that pertactin-deficient strains are better able to colonize the respiratory tract (*29*). According to some authors, immunization with 2-component ACVs (instead of an ACV with 4 or 5 components) might affect immunogenicity (*30*–*32*). However, more time is needed to evaluate whether the lack of the pertactin component of 1 of the ACVs affects the incidence of pertussis in Austria in the coming years. Nevertheless, the higher likelihood of profile B strains found in the St. Johann in Pongau district seems not to have been influenced by vaccination.

Regarding the mechanisms causing pertactin deficiency, we reported a mutation at the 632 nt of the *prn* gene for our profile B isolates, previously described for isolates collected in Italy, Sweden, and Denmark during 2012–2015 (*15*). Likewise, this deletion was reported in Ireland in 2016 in an isolate (GenBank accession no. KX462969.1) differing from the isolates of cluster 2 in Austria by only 8 alleles. On the contrary, the mutation T223C in profile H had been previously reported during 2012–2015 in Australia, the Netherlands, Norway, Sweden, the United Kingdom, and the United States (*33*–*35*). The mutation in the *prn* gene at nt 1326 was reported in the United Kingdom (*16*) in 2012. Similar mutations at nt 1325 and 1340 of the *prn* gene were also detected in isolates from Australia (*35*) and the United States (*36*). Likewise, the mutation at nt 1273 had been previously found in Canada (*37*) and the United States as well (*13*,*14*). Last, the insertion of the *IS*481 at nt 1613 in reverse direction was also reported in Canada and the United States (*13*,*14*).

The preliminary cluster threshold proposed in this study has served to delineate clusters and can be adjusted when more epidemiologic data derived from contact tracing are available. Establishing a fixed cluster threshold for *B. pertussis* is challenging also because of its homogeneous core genome. Also, because the bacterium undergoes large genomic rearrangements that are only detectable with advanced bioinformatics (*14*), this diversity might not be captured by cgMLST alone. A possibility to increase the typing resolution obtained with cgMLST for detecting pertussis outbreaks might be to investigate the distribution of *IS*481 within the *B. pertussis* genome, as proposed elsewhere (*14*). In either case, cgMLST allowed the identification of a cluster (cluster 2) of pertactin-deficient isolates from case-patients living in St. Johann in Pongau, possibly indicating the presence of a local pertussis outbreak. We could not determine whether all cases occurring within the period of detection of the genetic profile B belonged to that profile or to another genetic profile because we did not receive *B. pertussis* cultures for each pertussis case. Except for cluster 2, we could neither confirm nor refute that all clusters identified in our study represented single outbreaks. However, we demonstrated the direct transmission of the same pertussis strain by cgMLST among members of the same household. We hypothesize that the 2 case-patients in the household where 2 different genetic profiles and cgMLST (18 alleles of difference) were detected might have acquired pertussis from different sources.

Our results of the comparative genomic analysis using global strains were concordant with other studies, in which diverse *B. pertussis* genetic profiles are shown to be distributed across countries (*12*–*14*,*16*). The pertactin-deficient strains seemed not to belong to the same clone and the mutations observed in each country might consist of independent mutations, as previously described (*38*). In the absence of more sequences to compare with our isolates in Austria, profile B isolates seemed to be found only in Austria, although the mutation *prn*-2–631^632STOP:T>- had already been reported (*15*). In addition, the number of allelic differences between isolates not geographically nor temporally related to the isolates in Austria was sometimes as low as 2, matching recently reported findings (*12*).

The small differences observed between the SNP-based analysis and the cgMLST-based analysis might be attributable to the slightly higher discriminatory power of SNP-based analysis (*39*). Conversely, the differences in gene content between the Pasteur Institute’s cgMLST scheme and ours indicate that our cgMLST was more discriminatory and therefore more suitable for cluster detection in Austria. The uneven number of loci composing each cgMLST might be partially attributable to the different algorithms used by Seqsphere and BIGSdb (*40*) but also to the fact that only targets that were present in all query genomes were included as targets of our cgMLST scheme. In contrast, the Pasteur Institute’s scheme included targets present in >95% of all query genomes. Bouchez et al. (*12*) did not define a threshold for their cgMLST scheme, and hence both schemes might not be comparable in terms of isolate clustering.

The main limitation of our study was the incomplete information on the vaccination status of the case-patients and other epidemiologic data, which prevented better assessment of the effects of these genetic shifts on pertussis incidence. Because of the reduced sample size, whether detecting pertactin-deficient strains is linked to an increase in pertussis incidence is unclear; therefore, expanding the isolate-based surveillance system at the national level is advisable.

In summary, we found that *B. pertussis* strains in Austria differ genetically from the vaccine strain, both in their core genomes and their vaccine antigen genes. Furthermore, our cgMLST method has proven to be stable enough to be applied beyond our set of 123 *B. pertussis* isolates and proven useful to confirm transmission chains among household members and to detect 8 clusters, 1 of which indicated a possible local outbreak. To detect pertussis outbreaks and target pertussis-control strategies, we recommend performing genomic surveillance of *B. pertussis* using the proposed cgMLST scheme with a preliminary cluster threshold of <6 alleles, typing data on the vaccine antigen genes, and complete epidemiologic information on pertussis cases.

Appendix 1 Additional description of core-genome multilocus sequence typing conducted in isolate-based surveillance of *Bordetella pertussis*, Austria, 2018–2020.

Appendix 2 Additional data pertaining to core-genome multilocus sequence typing conducted in isolate-based surveillance of *Bordetella pertussis*, Austria, 2018–2020.

## References

[1] Kilgore PE, Salim AM, Zervos MJ, Schmitt HJ. Pertussis: microbiology, disease, treatment, and prevention. Clin Microbiol Rev. 2016;29:449–86. 10.1128/CMR.00083-15 27029594 PMC4861987

[2] Paisley RD, Blaylock J, Hartzell JD. Whooping cough in adults: an update on a reemerging infection. Am J Med. 2012;125:141–3. 10.1016/j.amjmed.2011.05.008 22269615

[3] Lee AD, Cassiday PK, Pawloski LC, Tatti KM, Martin MD, Briere EC, et al.; Clinical Validation Study Group. Clinical evaluation and validation of laboratory methods for the diagnosis of *Bordetella pertussis* infection: Culture, polymerase chain reaction (PCR) and anti-pertussis toxin IgG serology (IgG-PT). PLoS One. 2018;13:e0195979. 10.1371/journal.pone.0195979 29652945 PMC5898745

[4] Mir-Cros A, Moreno-Mingorance A, Martín-Gómez MT, Codina G, Cornejo-Sánchez T, Rajadell M, et al. Population dynamics and antigenic drift of *Bordetella pertussis* following whole cell vaccine replacement, Barcelona, Spain, 1986-2015. Emerg Microbes Infect. 2019;8:1711–20. 10.1080/22221751.2019.1694395 31769735 PMC6882445

[5] Wagner B, Melzer H, Freymüller G, Stumvoll S, Rendi-Wagner P, Paulke-Korinek M, et al. Genetic variation of *Bordetella pertussis* in Austria. PLoS One. 2015;10:e0132623. 10.1371/journal.pone.0132623 26182210 PMC4504479

[6] Latasa P, García-Comas L, Gil de Miguel A, Barranco MD, Rodero I, Sanz JC, et al. Effectiveness of acellular pertussis vaccine and evolution of pertussis incidence in the community of Madrid from 1998 to 2015. Vaccine. 2018;36:1643–9. 10.1016/j.vaccine.2018.01.070 29439872

[7] Esposito S, Stefanelli P, Fry NK, Fedele G, He Q, Paterson P, et al.; World Association of Infectious Diseases and Immunological Disorders (WAidid) and the Vaccine Study Group of the European Society of Clinical Microbiology and Infectious Diseases (EVASG). (WAidid) and the Vaccine Study Group of the European Society of Clinical Microbiology and Infectious Diseases (EVASG). Pertussis prevention: reasons for resurgence, and differences in the current acellular pertussis vaccines. Front Immunol. 2019;10:1344. 10.3389/fimmu.2019.01344 31333640 PMC6616129

[8] Burdin N, Handy LK, Plotkin SA. What is wrong with pertussis vaccine immunity? The problem of waning effectiveness of pertussis vaccines. Cold Spring Harb Perspect Biol. 2017;9:a029454. 10.1101/cshperspect.a029454 28289064 PMC5710106

[9] Chiappini E, Stival A, Galli L, de Martino M. Pertussis re-emergence in the post-vaccination era. BMC Infect Dis. 2013;13:151. 10.1186/1471-2334-13-151 23530907 PMC3623740

[10] Mooi FR, van Oirschot H, Heuvelman K, van der Heide HGJ, Gaastra W, Willems RJL. Polymorphism in the *Bordetella pertussis* virulence factors P.69/pertactin and pertussis toxin in The Netherlands: temporal trends and evidence for vaccine-driven evolution. Infect Immun. 1998;66:670–5. 10.1128/IAI.66.2.670-675.1998 9453625 PMC107955

[11] Diavatopoulos DA, Cummings CA, Schouls LM, Brinig MM, Relman DA, Mooi FR. *Bordetella pertussis*, the causative agent of whooping cough, evolved from a distinct, human-associated lineage of *B. bronchiseptica.* PLoS Pathog. 2005;1:e45. 10.1371/journal.ppat.0010045 16389302 PMC1323478

[12] Bouchez V, Guglielmini J, Dazas M, Landier A, Toubiana J, Guillot S, et al. Genomic sequencing of *Bordetella pertussis* for epidemiology and global surveillance of whooping cough. Emerg Infect Dis. 2018;24:988–94. 10.3201/eid2406.171464 29774847 PMC6004856

[13] Bowden KE, Weigand MR, Peng Y, Cassiday PK, Sammons S, Knipe K, et al. Genome structural diversity among 31 *Bordetella pertussis* isolates from two recent U.S. whooping cough statewide epidemics. MSphere. 2016;1:e00036–16. 10.1128/mSphere.00036-16 27303739 PMC4888882

[14] Weigand MR, Peng Y, Loparev V, Batra D, Bowden KE, Burroughs M, et al. The history of *Bordetella pertussis* genome evolution includes structural rearrangement. J Bacteriol. 2017;199:199. 10.1128/JB.00806-16 28167525 PMC5370423

[15] Barkoff A-M, Mertsola J, Pierard D, Dalby T, Hoegh SV, Guillot S, et al. Pertactin-deficient *Bordetella pertussis* isolates: evidence of increased circulation in Europe, 1998 to 2015. Euro Surveill. 2019;24:1700832. 10.2807/1560-7917.ES.2019.24.7.1700832 30782265 PMC6381657

[16] Sealey KL, Harris SR, Fry NK, Hurst LD, Gorringe AR, Parkhill J, et al. Genomic analysis of isolates from the United Kingdom 2012 pertussis outbreak reveals that vaccine antigen genes are unusually fast evolving. J Infect Dis. 2015;212:294–301. 10.1093/infdis/jiu665 25489002

[17] Vestrheim DF, Steinbakk M, Bjørnstad ML, Moghaddam A, Reinton N, Dahl ML, et al. Recovery of *Bordetella pertussis* from PCR-positive nasopharyngeal samples is dependent on bacterial load. J Clin Microbiol. 2012;50:4114–5. 10.1128/JCM.01553-12 23035189 PMC3502974

[18] Martini H, Rodeghiero C, VAN DEN Poel C, Vincent M, Pierard D, Huygen K. Pertussis diagnosis in Belgium: results of the National Reference Centre for Bordetella anno 2015. Epidemiol Infect. 2017;145:2366–73. 10.1017/S0950268817001108 28578723 PMC9148849

[19] Lam C, Octavia S, Bahrame Z, Sintchenko V, Gilbert GL, Lan R. Selection and emergence of pertussis toxin promoter *ptxP3* allele in the evolution of *Bordetella pertussis.* Infect Genet Evol. 2012;12:492–5. 10.1016/j.meegid.2012.01.001 22293463

[20] Bowden KE, Williams MM, Cassiday PK, Milton A, Pawloski L, Harrison M, et al. Molecular epidemiology of the pertussis epidemic in Washington State in 2012. J Clin Microbiol. 2014;52:3549–57. 10.1128/JCM.01189-14 25031439 PMC4187741

[21] van Gent M, Heuvelman CJ, van der Heide HG, Hallander HO, Advani A, Guiso N, et al. Analysis of *Bordetella pertussis* clinical isolates circulating in European countries during the period 1998-2012. Eur J Clin Microbiol Infect Dis. 2015;34:821–30. 10.1007/s10096-014-2297-2 25527446 PMC4365279

[22] Bart MJ, Harris SR, Advani A, Arakawa Y, Bottero D, Bouchez V, et al. Global population structure and evolution of *Bordetella pertussis* and their relationship with vaccination. MBio. 2014;5:e01074–14. 10.1128/mBio.01074-14 24757216 PMC3994516

[23] Moriuchi T, Vichit O, Vutthikol Y, Hossain MS, Samnang C, Toda K, et al. Molecular epidemiology of *Bordetella pertussis* in Cambodia determined by direct genotyping of clinical specimens. Int J Infect Dis. 2017;62:56–8. 10.1016/j.ijid.2017.07.015 28751008

[24] Miyaji Y, Otsuka N, Toyoizumi-Ajisaka H, Shibayama K, Kamachi K. Genetic analysis of *Bordetella pertussis* isolates from the 2008-2010 pertussis epidemic in Japan. PLoS One. 2013;8:e77165. 10.1371/journal.pone.0077165 24124606 PMC3790747

[25] Breakwell L, Kelso P, Finley C, Schoenfeld S, Goode B, Misegades LK, et al. Pertussis vaccine effectiveness in the setting of pertactin-deficient pertussis. Pediatrics. 2016;137:e20153973. 10.1542/peds.2015-3973 27244813

[26] Etskovitz H, Anastasio N, Green E, May M. Role of evolutionary selection scting on vaccine antigens in the re-emergence of *Bordetella pertussis.* Diseases. 2019;7:35. 10.3390/diseases7020035 30995764 PMC6630436

[27] Safarchi A, Octavia S, Nikbin VS, Lotfi MN, Zahraei SM, Tay CY, et al. Genomic epidemiology of Iranian *Bordetella pertussis*: 50 years after the implementation of whole cell vaccine. Emerg Microbes Infect. 2019;8:1416–27. 10.1080/22221751.2019.1665479 31543006 PMC6764348

[28] Carriquiriborde F, Regidor V, Aispuro PM, Magali G, Bartel E, Bottero D, et al. Rare detection of *Bordetella pertussis* pertactin-deficient strains in Argentina. Emerg Infect Dis. 2019;25:2048–54. 10.3201/eid2511.190329 31625838 PMC6810201

[29] Safarchi A, Octavia S, Luu LD, Tay CY, Sintchenko V, Wood N, et al. Pertactin negative *Bordetella pertussis* demonstrates higher fitness under vaccine selection pressure in a mixed infection model. Vaccine. 2015;33:6277–81. 10.1016/j.vaccine.2015.09.064 26432908

[30] Carvalho CFA, Andrews N, Dabrera G, Ribeiro S, Stowe J, Ramsay M, et al. National Outbreak of Pertussis in England, 2011–2012: a case-control study comparing 3-component and 5-component acellular vaccines with whole-cell pertussis vaccines. Clin Infect Dis. 2020;70:200–7. 10.1093/cid/ciz199 31059566

[31] van Twillert I, Bonačić Marinović AA, Kuipers B, van Gaans-van den Brink JAM, Sanders EAM, van Els CACM. Impact of age and vaccination history on long-term serological responses after symptomatic *B. pertussis* infection, a high dimensional data analysis. Sci Rep. 2017;7:40328. 10.1038/srep40328 28091579 PMC5238437

[32] Olin P, Rasmussen F, Gustafsson L, Hallander HO, Heijbel H; Ad Hoc Group for the Study of Pertussis Vaccines. Randomised controlled trial of two-component, three-component, and five-component acellular pertussis vaccines compared with whole-cell pertussis vaccine. Lancet. 1997;350:1569–77. 10.1016/S0140-6736(97)06508-2 9393335

[33] Zeddeman A, van Gent M, Heuvelman CJ, van der Heide HG, Bart MJ, Advani A, et al. Investigations into the emergence of pertactin-deficient *Bordetella pertussis* isolates in six European countries, 1996 to 2012. Euro Surveill. 2014;19:20881. 10.2807/1560-7917.ES2014.19.33.20881 25166348

[34] Weigand MR, Williams MM, Peng Y, Kania D, Pawloski LC, Tondella ML; CDC Pertussis Working Group. Genomic survey of *Bordetella pertussis* diversity, United States, 2000–2013. Emerg Infect Dis. 2019;25:780–3. 10.3201/eid2504.180812 30882317 PMC6433035

[35] Xu Z, Octavia S, Luu LDW, Payne M, Timms V, Tay CY, et al. Pertactin-negative and filamentous hemagglutinin-negative *Bordetella pertussis*, Australia, 2013–2017. Emerg Infect Dis. 2019;25:1196–9. 10.3201/eid2506.180240 31107218 PMC6537726

[36] Weigand MR, Peng Y, Cassiday PK, Loparev VN, Johnson T, Juieng P, et al. Complete genome sequences of *Bordetella pertussis* isolates with novel pertactin-deficient deletions. Genome Announc. 2017;5:e00973–17. 10.1128/genomeA.00973-17 28912323 PMC5597764

[37] Tsang RS, Shuel M, Jamieson FB, Drews S, Hoang L, Horsman G, et al. Pertactin-negative *Bordetella pertussis* strains in Canada: characterization of a dozen isolates based on a survey of 224 samples collected in different parts of the country over the last 20 years. Int J Infect Dis. 2014;28:65–9. 10.1016/j.ijid.2014.08.002 25244999

[38] Lam C, Octavia S, Ricafort L, Sintchenko V, Gilbert GL, Wood N, et al. Rapid increase in pertactin-deficient *Bordetella pertussis* isolates, Australia. Emerg Infect Dis. 2014;20:626–33. 10.3201/eid2004.131478 24655754 PMC3966384

[39] Uelze L, Grützke J, Borowiak M, Hammerl JA, Juraschek K, Deneke C, et al. Typing methods based on whole genome sequencing data. One Health Outlook. 2020;2:3. 10.1186/s42522-020-0010-1 33829127 PMC7993478

[40] Jolley KA, Bray JE, Maiden MCJ. Open-access bacterial population genomics: BIGSdb software, the PubMLST.org website and their applications. Wellcome Open Res. 2018;3:124. 10.12688/wellcomeopenres.14826.1 30345391 PMC6192448

