# Supplementary material for: Isolate-Based Surveillance of Bordetella pertussis, Austria, 2018–2020
Source: Emerg Infect Dis. 2021 Mar;27(3):862–71. doi: 10.3201/eid2703.202314 (PMC7920692; doi:10.3201/eid2703.202314)
Supplement: Appendix 1 — Additional description of core-genome multilocus sequence typing conducted in isolate-based surveillance of Bordetella pertussis, Austria, 2018–2020. [file 20-2314-Techapp-s1.pdf]

Article DOI: https://doi.org/10.3201/eid2703.202314

Isolate-based surveillance of *Bordetella pertussis*, Austria, 2018–2020

Appendix 1

MLST Scheme

We obtained the seven *B. pertussis* MLST loci sequences and their allelic variants from an existing *Bordetella sp.* scheme (*1*) to type our Austrian isolates.

Definition of the cgMLST Scheme and Validation

We generated with Ridom SeqSphere+ software version 4.1.9., a stable cgMLST scheme for ad hoc usage containing all genes within the reference genome but the non-homologous, those with internal stop codons and those that overlapped other genes. Briefly, the reference genome (now called representative genome) Tohama I (NC_002929.2) was selected as a seed genome for the cgMLST using the *target definer* function of SeqSphere and applying the following filters:

1. A minimum length filter, which required a minimum of 50 bases.
2. A start codon filter, which required the presence of a start codon at beginning of each gene.
3. A stop codon filter, which required the presence of a single stop codon at end of gene. Two targets were discarded: BP1104, BP2738.
4. A homologous gene filter, which required the presence of no more than one gene copy with a BLAST (*2*) overlap of ≥100bp and an identity of ≥90.0%. 261 targets were discarded: BP0007, BP0023, BP0031, BP0041, BP0049, BP0058, BP0071, BP0080, BP0110, BP0118, BP0124, BP0137, BP0166, BP0175, BP0192, BP0202, BP0203, BP0210, BP0211, BP0228, BP0256, BP0268, BP0281, BP0295, BP0297, BP0327, BP0355, BP0365, BP0392, BP0401, BP0408,

BP0424, BP0439, BP0443, BP0473, BP0481, BP0496, BP0514, BP0517, BP0537, BP0540, BP0565, BP0579, BP0581, BP0582, BP0596, BP0597, BP0611, BP0645, BP0646, BP0676, BP0688, BP0704, BP0716, BP0729, BP0733, BP0739, BP0785, BP0786, BP0797, BP0812, BP0830, BP0838, BP0839, BP0867, BP0871, BP0891, BP0897, BP0910, BP0938, BP0957, BP0994, BP0995, BP0997, BP1020, BP1030, BP1035, BP1044, BP1048, BP1053, BP1064, BP1067, BP1080, BP1086, BP1093, BP1118, BP1130, BP1134, BP1142, BP1157, BP1177, BP1199, BP1201, BP1224, BP1268, BP1278, BP1287, BP1308, BP1332, BP1337, BP1361, BP1365, BP1384, BP1385, BP1388, BP1397, BP1439, BP1450, BP1459, BP1488, BP1489, BP1491, BP1493, BP1511, BP1544, BP1552, BP1557, BP1572, BP1594, BP1602, BP1629, BP1630, BP1633, BP1647, BP1653, BP1656, BP1678, BP1689, BP1697, BP1711, BP1717, BP1735, BP1748, BP1757, BP1792, BP1807, BP1809, BP1810, BP1844, BP1866, BP1879, BP1911, BP1914, BP1947, BP1955, BP1957, BP1958, BP1959, BP2018, BP2029, BP2048, BP2054, BP2087, BP2104, BP2105, BP2121, BP2135, BP2137, BP2166, BP2181, BP2207, BP2214, BP2221, BP2266, BP2272, BP2297, BP2316, BP2355, BP2390, BP2415, BP2453, BP2477, BP2485, BP2492, BP2524, BP2568, BP2577, BP2579, BP2582, BP2587, BP2608, BP2630, BP2666, BP2667, BP2672, BP2673, BP2679, BP2704, BP2721, BP2724, BP2733, BP2748, BP2763, BP2776, BP2781, BP2812, BP2819, BP2821, BP2845, BP2848, BP2852, BP2861, BP2884, BP2912, BP2947, BP2955, BP2976, BP3005, BP3046, BP3049, BP3055, BP3065, BP3091, BP3103, BP3111, BP3114, BP3149, BP3150, BP3164, BP3185, BP3186, BP3203, BP3210, BP3216, BP3220, BP3230, BP3243, BP3257, BP3272, BP3279, BP3294, BP3311, BP3312, BP3313, BP3323, BP3336, BP3386, BP3392, BP3406, BP3408, BP3423, BP3436, BP3451, BP3456, BP3478, BP3505, BP3510, BP3519, BP3520, BP3548, BP3593, BP3603, BP3607, BP3611, BP3698, BP3726, BP3806, BP3810, BP3811, BP3839, BP3851.

5. A gene overlap filter, which required no overlap between a core gene with other genes by more than 4 bases. Ninety-seven targets were filtered and moved to the

accessory genome scheme: BP0036, BP0044, BP0144, BP0169, BP0305, BP0320, BP0417, BP0437, BP0504, BP0619, BP0656, BP0684A, BP0799, BP0836, BP0946, BP1091, BP1106, BP1108, BP1136, BP1159, BP1182, BP1190, BP1344, BP1356, BP1393, BP1399, BP1401, BP1402, BP1433, BP1442, BP1591, BP1639, BP1650, BP1655, BP1665, BP1733, BP1754, BP1789, BP1825, BP1837, BP1883, BP1993, BP2061, BP2080, BP2107, BP2130, BP2150, BP2161, BP2192, BP2236, BP2246, BP2247, BP2264, BP2311, BP2320, BP2328, BP2330, BP2350, BP2381, BP2429, BP2475, BP2603, BP2612, BP2652, BP2658, BP2686, BP2710, BP2729, BP2878, BP2880, BP2902, BP2965, BP3042, BP3155, BP3188, BP3190, BP3277, BP3325, BP3349, BP3357, BP3363, BP3385, BP3389, BP3433, BP3517, BP3556, BP3770, BP3785, BP3792, BP3794, BP3796, BP3799, BP3801, BP3808, BP3824, BP3864, BP3865.

Afterwards we selected 15 publicly accessible *B. pertussis* genomes as query genomes as of 2nd of February 2018 (Appendix 2 Table 1). The selection was based on the center where the genome was sequenced and release date, so that the scheme contained *B. pertussis* strains obtained in different countries and years, avoiding over representing certain countries with more sequences available in NCBI. First, a query genome BLAST (version 2.2.12) search was performed. This required a BLAST hit with an overlap of 100% and an identity of ≥90.0% in every query genome. The following BLAST options were set: Mismatch penalty = −1, match reward = 1, gap open costs = 5, gap extension costs = 2. This query genome BLAST search filtered out 81 targets and all were moved to the accessory genome scheme: BP0184, BP0200, BP0422, BP0515, BP0593, BP0594, BP0635, BP0711, BP0911, BP0913, BP0914, BP0915, BP0918, BP0919, BP0920, BP0921, BP0922, BP0923, BP0924, BP0925, BP0926, BP0927, BP0928, BP0929, BP0930, BP0931, BP0932, BP0934, BP1014, BP1015, BP1016, BP1017, BP1018, BP1019, BP1054, BP1137, BP1138, BP1139, BP1141, BP1174, BP1323, BP1592, BP1948, BP1949, BP1950, BP1951, BP1953, BP1954, BP1961, BP1962, BP1965, BP1987, BP2075, BP2138, BP2139, BP2268, BP2369, BP2451, BP2452, BP2455, BP2820, BP2946, BP2990, BP3105, BP3106, BP3107, BP3108, BP3109, BP3110, BP3115, BP3160, BP3314, BP3315, BP3316, BP3317, BP3319, BP3320, BP3321, BP3322, BP3663, BP3764.

In addition, a stop codon percentage filter was applied to the query genomes (n = 15). This filter required a single stop codon at end of a gene in >80% of the query genomes. One target was filtered and moved to the accessory genome scheme: BP1123.

In summary, 2,983 targets (Appendix 2 Table 2) were defined for cgMLST with a total of 2,932,632 bases, 179 targets were used as accessory targets (Appendix 2 Table 3) with 159,468 bases and 263 targets were discarded. Of the reference genome NC_002929.2 71.8% bases were covered by cgMLST targets. Between 70.9% and 71.45% of query genomes bases were covered by cgMLST targets. Only targets present in all query genomes were included as targets of the final cgMLST scheme ("hard core" cgMLST scheme).

Further evaluation of the scheme included the addition of all available assembled *B. pertussis* genomes from NCBI (n = 537) as of 2nd February 2018. We manually discarded genomes with low WGS quality data. Finally, a collection of 359 *B. pertussis* genomes with a diverse genetic background (Appendix 2 Table 4) remained. All but two genomes presented more than 95% good targets. Afterwards, we also included two non-pertussis strains and one *B. pertussis* strain from an old Austrian isolate collection (Appendix 2 Table 5) and 32 genomes using Illumina reads from SRA (Appendix 2 Table 4).

Sequencing and de novo Assembly of the Austrian *B. pertussis* Isolates

DNA isolation from *B. pertussis* cultures was performed with MagAttract HMW DNA kit (Qiagen, Hilden, Germany) and quantified with Qubit version 2.0 fluorometer (Thermo Fisher Scientific, Waltham, MA, USA) using the double-stranded DNA broad-range (dsDNA BR) assay kit (Thermo Fisher Scientific). Library preparation was performed using Nextera XT Kit (Illumina, San Diego, USA) followed by 300bp paired-end sequencing on an Illumina Miseq platform. Raw reads were de novo assembled with SPAdes version 3.11.1 (*3*). using default parameters. QUAST v5.0.2 (*4*) was used to evaluate the quality of the genome assemblies by extracting information on the N50, the number of contigs and the assembly length for each genome. Additionally, when pangenome assemblies were detected we used CheckM v1.1.2 (*5*) to assess the completeness and contamination level of the sequences (Appendix 2 Table 6).

Typing of the Austrian *B. pertussis* Isolates

Isolates were typed with our newly implemented cgMLST and accessory genome schemes using SeqSphere+, which uses BLAST version 2.2.12 and ignores *contigs* shorter than 200 bases. A target quality control (QC) procedure was included in all typing schemes. This QC required: 1) the length of the consensus being the same as the ref.-seq. area(s) length +-3 triplets, 2) no ambiguities (R,Y,K,M,S,W,B,D,H,V,N) in the consensus area(s) 3) no frame shift in the translatable consensus area(s). Afterwards, the software scanned with BLASTN the sequences of each scheme with the following parameters: mismatch = −1, match = 1, gap open = 5, gap extension = 2 and threshold for each hit of ≥90% identity and 100% aligned to the reference sequence. Isolates was re-sequenced when the percentage of "good targets" referring to the cgMLST scheme was inferior to 95%. To construct phylogenetic trees we always ignored missing values pairwise.

The cluster threshold was preliminary set at 6 allelic differences. We took into consideration the number of allelic differences between the 123 Austrian isolates (Figure 3, https://wwwnc.cdc.gov/EID/article/27/3/20-2314-F3.htm), the available epidemiologic data for the Austrian cases, such as district of residence or cohabitation, and the combination of allelic variants and mutations detected in the vaccine antigen genes *ptx*S1, *ptx*P, *prn*, *fim*2 and *fim*3 (here called as "genetic profiles").

Other Typing Schemes

We extracted the sequences of the *B. pertussis* vaccine antigen genes from the WGS data. To do so, we downloaded from PubMLST all those FASTA sequences comprising all the available allelic variants for the vaccine antigen genes until 2nd February 2018 (https://pubmlst.org/bigsdb?db = pubmlst_bordetella_seqdef&page = downloadAlleles&tree = 1) . Afterwards we generated an allele library typing scheme including the vaccine antigen genes *ptx*S1, *ptx*P, *prn*, *fim*2 and *fim*3 and their variants. The typing schemes were configured to detect new alleles for each of the targets with at least 90% identity and 98% overlap to the reference alleles.

Detection of PRN-Deficient Isolates

When the *prn* gene was not found using Seqsphere+, we first made a BLAST using the assembly file and the *prn* gene sequence from Tohama I (NC_002929.2). If two different fragments of the pertactin gene were present in at least two different *contigs*, the *prn* gene was most probably truncated by an insertion sequence. In this case, we mapped the raw reads against the *B. pertussis* Tohama I reference strain using the Burrows–Wheeler Aligner (BWA-MEM) version 0.7.16a-r1181 (*6*) to check whether these truncations in the *prn* gene were an artifact from the assembly process or a real genetic modification. Afterwards, we used PRODIGAL v2.6.3 (*7*) and BLAST 2.10.0+ to obtain the annotations and the predicted genes with tags on them. Lastly, the resultant BAM files were visualized using Tablet v1.19.09.03 (*8*) to distinguish possible areas near or within the *prn* gene with an absence of mapped reads. If no read pairs spanned the insertion sites, an insertion of >200 nt was suspected.

When the pertactin gene sequence differed from the alleles in the *prn* typing scheme, Seqsphere marked the target as “failed” or “new,” indicating the presence of a genetic modification (e.g.: insertion, deletion and/or base change, with or without a stop codon).

SNP Analysis

The cgMLST-based analysis on the 123 Austrian *B. pertussis* assembled genomes was compared with a SNP-based approach (Appendix 1 Figure 2) using a custom script for variant calling. First, the paired-end reads were trimmed with Trimmomatic (*9*) version 0.39 using the default parameters and then mapped against the reference genome Tohama I (NC_002929.2) using BWA-MEM. SNPs were called using the *mpileup* and *call* commands from bcftools version 1.6–14- geed5371 with default parameters (*10*). The SNPs were then filtered with the vcfutils.pl varFilter script available in SAMtools version 1.11 and indels were excluded with VCFtools 0.1.16 (*11*,*12*). Afterwards the SNPs in all genomes were concatenated in a single fasta alignment. We further inferred the phylogeny with VCF-toolkit version 0.1.6 (*13*). The resultant neighbor-joining tree was compared to the one obtained using the core genome with the Tanglegram option in Dendroscope version 3.7.2 (*14*).

References

1. Diavatopoulos DA, Cummings CA, Schouls LM, Brinig MM, Relman DA, Mooi FR. *Bordetella pertussis*, the causative agent of whooping cough, evolved from a distinct, human-associated lineage of *B. bronchiseptica*. PLoS Pathog. 2005;1:e45. PubMed https://doi.org/10.1371/journal.ppat.0010045

2. Camacho C, Coulouris G, Avagyan V, Ma N, Papadopoulos J, Bealer K, et al. BLAST+: architecture and applications. BMC Bioinformatics. 2009;10:421. PubMed https://doi.org/10.1186/1471-2105-10-421

3. Bankevich A, Nurk S, Antipov D, Gurevich AA, Dvorkin M, Kulikov AS, et al. SPAdes: a new genome assembly algorithm and its applications to single-cell sequencing. J Comput Biol. 2012;19:455–77. PubMed https://doi.org/10.1089/cmb.2012.0021

4. Gurevich A, Saveliev V, Vyahhi N, Tesler G. QUAST: quality assessment tool for genome assemblies. Bioinformatics. 2013;29:1072–5. PubMed https://doi.org/10.1093/bioinformatics/btt086

5. Parks DH, Imelfort M, Skennerton CT, Hugenholtz P, Tyson GW. CheckM: assessing the quality of microbial genomes recovered from isolates, single cells, and metagenomes. Genome Res. 2015;25:1043–55. PubMed https://doi.org/10.1101/gr.186072.114

6. Li H, Durbin R. Fast and accurate short read alignment with Burrows-Wheeler transform. Bioinformatics. 2009;25:1754–60. PubMed https://doi.org/10.1093/bioinformatics/btp324

7. Hyatt D, Chen G-L, Locascio PF, Land ML, Larimer FW, Hauser LJ. Prodigal: prokaryotic gene recognition and translation initiation site identification. BMC Bioinformatics. 2010;11:119. PubMed https://doi.org/10.1186/1471-2105-11-119

8. Milne I, Bayer M, Cardle L, Shaw P, Stephen G, Wright F, et al. Tablet—next generation sequence assembly visualization. Bioinformatics. 2010;26:401–2. PubMed https://doi.org/10.1093/bioinformatics/btp666

9. Bolger AM, Lohse M, Usadel B. Trimmomatic: a flexible trimmer for Illumina sequence data. Bioinformatics. 2014;30:2114–20. PubMed https://doi.org/10.1093/bioinformatics/btu170

10. Li H. A statistical framework for SNP calling, mutation discovery, association mapping and population genetical parameter estimation from sequencing data. Bioinformatics. 2011;27:2987–93. PubMed https://doi.org/10.1093/bioinformatics/btr509

11. Li H, Handsaker B, Wysoker A, Fennell T, Ruan J, Homer N, et al.; 1000 Genome Project Data Processing Subgroup. The Sequence Alignment/Map format and SAMtools. Bioinformatics. 2009;25:2078–9. PubMed https://doi.org/10.1093/bioinformatics/btp352

12. Danecek P, Auton A, Abecasis G, Albers CA, Banks E, DePristo MA, et al.; 1000 Genomes Project Analysis Group. The variant call format and VCFtools. Bioinformatics. 2011;27:2156–8. PubMed https://doi.org/10.1093/bioinformatics/btr330

13. Cook DE, Andersen EC. VCF-kit: assorted utilities for the variant call format. Bioinformatics. 2017;33:1581–2. PubMed https://doi.org/10.1093/bioinformatics/btx011

14. Huson DH, Richter DC, Rausch C, Dezulian T, Franz M, Rupp R. Dendroscope: an interactive viewer for large phylogenetic trees. BMC Bioinformatics. 2007;8:460. PubMed https://doi.org/10.1186/1471-2105-8-460

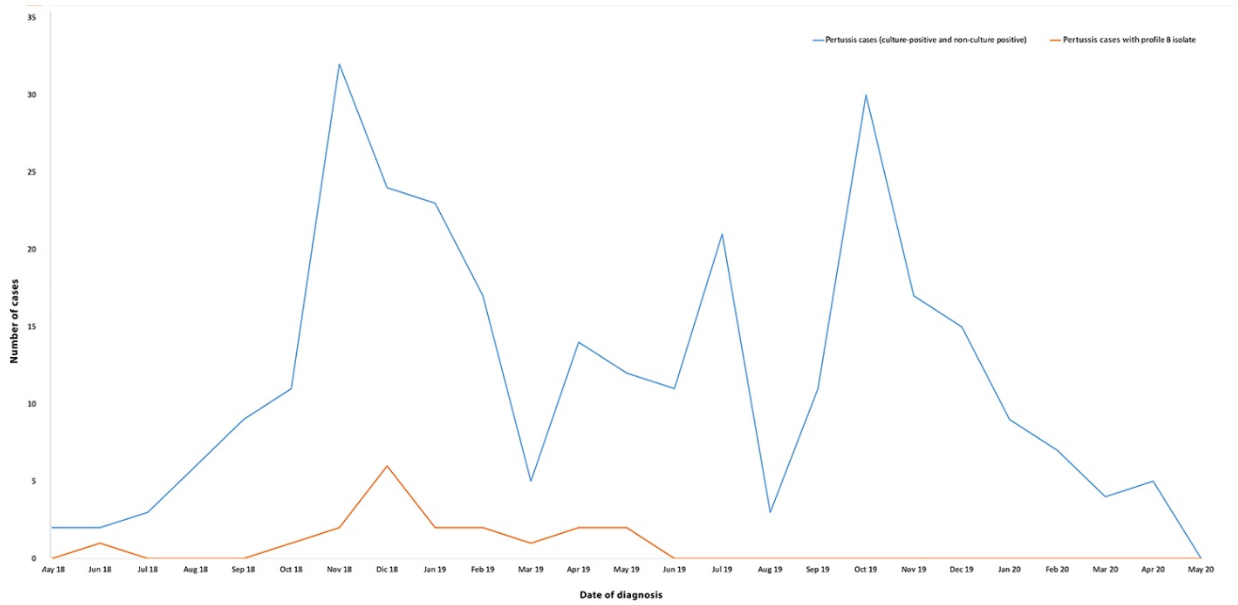

Appendix Figure 1. Cases with a genetic Profile B isolate (orange) vs. total pertussis cases (blue) detected in Sankt Johann im Pongau between May 2018 and May 2020. The total number of pertussis cases included both non-culture positive pertussis cases, for which no isolate was recovered and culture-positive cases of all genetic profiles, including Profile B.

Appendix 1 Figure 1. Cases with a genetic profile B isolate (orange) vs. total pertussis cases (blue) detected in Sankt Johann im Pongau between May 2018 and May 2020. The total number of pertussis cases included both culture-positive and non-culture positive pertussis cases (no isolate recovered) of any genetic profile, including profile B.

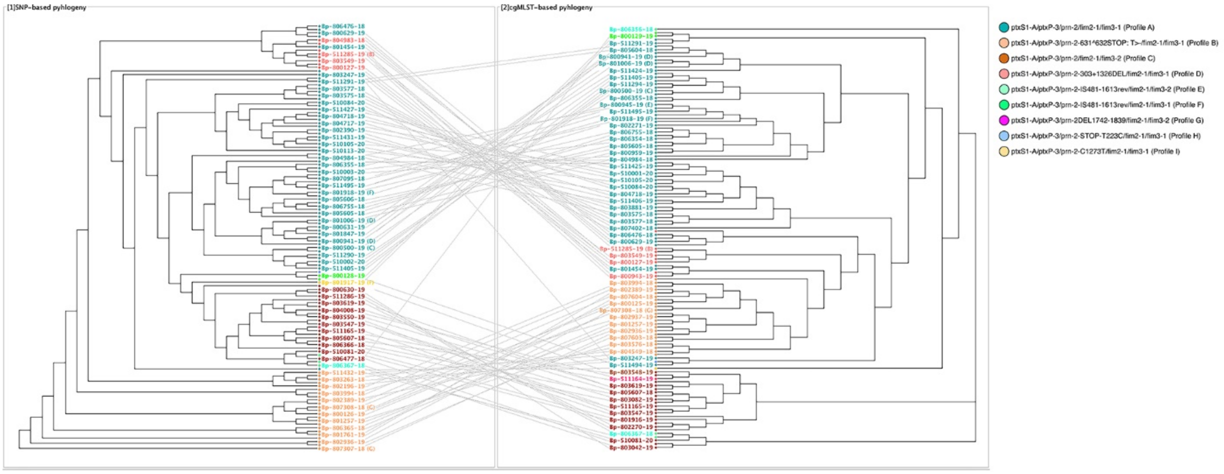

Appendix 1 Figure 2. Comparison of SNP (left) and cgMLST-based phylogenies (right) of the 123 Austrian *B. pertussis* isolates. Genetic profiles (A-I) are depicted in different colors. Letters (A-G) between parenthesis indicate households in which more than one pertussis case was detected. Due to limitations of the software, only a reduced number of isolates is displayed.
